# Molecularly Imprinted Polymer Based Sensors for Medical Applications

**DOI:** 10.3390/s19061279

**Published:** 2019-03-13

**Authors:** Yeşeren Saylan, Semra Akgönüllü, Handan Yavuz, Serhat Ünal, Adil Denizli

**Affiliations:** 1 Department of Chemistry, Hacettepe University, 06800 Ankara, Turkey; yeseren@hacettepe.edu.tr (Y.S.); semraakgonullu@hacettepe.edu.tr (S.A.); handany@hacettepe.edu.tr (H.Y.); 2 Department of Infectious Disease and Clinical Microbiology, Hacettepe University, 06230 Ankara, Turkey; sunal@hacettepe.edu.tr

**Keywords:** medical applications, medical sensors, molecular imprinting

## Abstract

Sensors have been extensively used owing to multiple advantages, including exceptional sensing performance, user-friendly operation, fast response, high sensitivity and specificity, portability, and real-time analysis. In recent years, efforts in sensor realm have expanded promptly, and it has already presented a broad range of applications in the fields of medical, pharmaceutical and environmental applications, food safety, and homeland security. In particular, molecularly imprinted polymer based sensors have created a fascinating horizon for surface modification techniques by forming specific recognition cavities for template molecules in the polymeric matrix. This method ensures a broad range of versatility to imprint a variety of biomolecules with different size, three dimensional structure, physical and chemical features. In contrast to complex and time-consuming laboratory surface modification methods, molecular imprinting offers a rapid, sensitive, inexpensive, easy-to-use, and highly selective approaches for sensing, and especially for the applications of diagnosis, screening, and theranostics. Due to its physical and chemical robustness, high stability, low-cost, and reusability features, molecularly imprinted polymer based sensors have become very attractive modalities for such applications with a sensitivity of minute structural changes in the structure of biomolecules. This review aims at discussing the principle of molecular imprinting method, the integration of molecularly imprinted polymers with sensing tools, the recent advances and strategies in molecular imprinting methodologies, their applications in medical, and future outlook on this concept.

## 1. Introduction

The sensing field represents an emerging technology with high capabilities and versatility to detect various analytes in different matrices and detection performance acts a significant function in several basic procedures in many systems [1,2]. Natural recognition elements have high affinity to their targets but they cannot be used in practical applications because of their poor durability at high pressure, temperature and in organic solvents, and also low stability in high and/or low pH buffers. Recently, one of the most important modification method, molecular imprinting, has been proposed to defeat most of these drawbacks [3]. This method was first reported by Wulff and Sarhan in the early 1970s [4]. After then, many of the researchers from all over the world observed the prospering of this method.

Molecular imprinting method principally bases on the specific molecular recognition to a template molecule [5]. Molecularly imprinted polymers (MIPs) can be synthesized by different types and combinations of functional monomers, cross-linkers, initiators and solvents [6,7,8]. The quality and feature of the imprinted polymers can be changed by the combination of the monomer and cross-linker mixtures, the experimental circumstances, the interaction mechanisms, and so on [9,10,11,12]. MIPs have multiple advantages including easy and cost-friendly preparation, high stability, high affinity and selectivity toward template molecule. In addition, MIPs can be employed in different areas, such as sensors [13,14], enzyme mimics [15,16], solid phase extraction [17], antibody mimics [18], enantioselective [19], biomarker [20,21] protein recognition and purification [22,23], food safety [24], sensing of microorganisms [25], drug analysis [26,27,28], drug delivery system [29], hormone detoxification [30], food and environmental analysis [31]. Sensors are self-contained integrated devices which are able to convert chemical signal depending on analyte concentration into a measurable analytical signal [32,33]. Sensors consist of two main components that are the receptor and the transducer. The receptor recognizes the analyte and the transducer is responsible for converting the binding event into a measurable signal [34]. Sensors possess many advantages such as selectivity, sensitivity, portability and quick response time; thereby, which are fabricated for various purposes especially for medical applications [35,36,37].

In this review, current trends in the development of molecularly imprinted polymer based sensor technology for rapid assessment of the medical applications, as well as future research directions were comprehensively discussed. Research findings showed that the use of molecularly imprinted polymer based sensors to detect biomolecules can improve the quality of the medical applications.

## 2. Molecular Imprinting Method

**Figure 1:**
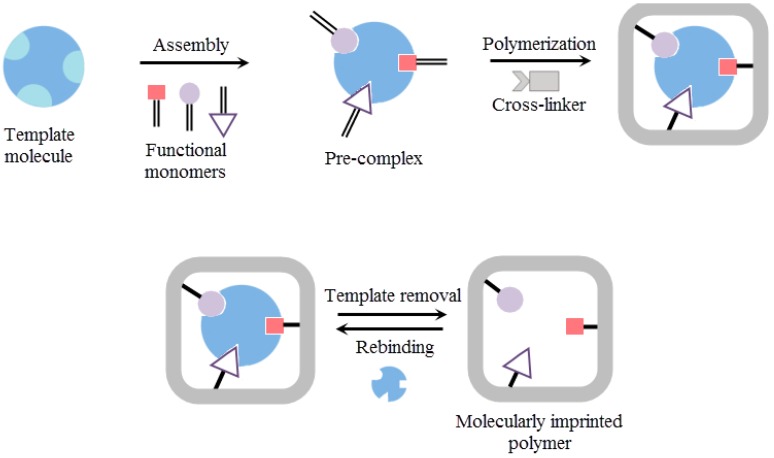
Scheme of the principle of molecularly imprinted polymer preparation.

A molecular imprinting method is based on the polymerization of a functional monomer and a cross-linker around a template molecule. At first, a pre-complex is formed between a template molecule and a functional monomer and then the polymerization is carried out around the pre-complex with initiator and cross-linker addition [38,39,40]. Finally, the template molecule is removed to generate three-dimensional cavities for specific recognition in several times (Figure 1).

**Figure 2:**
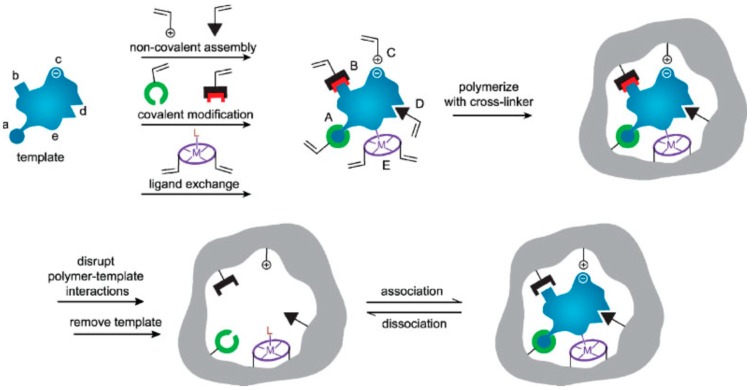
The principle of molecular imprinting recognition. Republished with permission from [42]; permission conveyed through the Copyright Clearance Center, Inc.

The template molecules, functional monomers, cross-linkers and initiators are necessary in optimum ratios for the complete polymerization. After the polymerization, highly specific recognition cavities are formed in the molecularly imprinted polymeric matrix for template molecules. MIPs can be prepared by different methods. As demonstrated in Figure 2, reversible covalent bonds (A), covalently attached polymerizable binding groups that are activated for non-covalent interactions (B), electrostatic interactions (C), hydrophobic or van der Waals interactions (D) or co-ordination with a metal center (E). All interactions are formed with complementary functional groups of the template molecules (a–e) [41,42].

In a molecularly imprinted polymer, specific sites can be created for various templates such as amino acids [43], proteins [44,45,46], enzymes [47,48], hormones [49,50,51], antibodies [52,53,54], nucleic acids [55,56], bacteria [57], viruses [58,59,60], drugs [61,62], metal ions [63,64,65,66], toxins [67], antibiotics [68] and pesticides [69,70] and so on. Furthermore, MIPs are easy to synthesis, highly stable, cost-effective, and user-friendly. They can be produced in large quantities with high reusability performance. These perfect recognition sites are interesting materials that are useful in especially medical applications [71].

## 3. Molecularly Imprinted Polymer Based Sensors

The detection of clinically relevant biomolecules is necessary to understand their biological and physiological functions and also to develop medical devices. The clinically relevant biomolecules perform several biological functions, such as genetic information storage and transmittance, biological activity regulation, biomolecules transportation, and reactions catalysis. In addition, they can be employed as biomarkers for diagnosis of many diseases [72]. Analytical devices, called sensors, consist of a transducer and recognition elements to capture an analyte for their structure evaluation and they act by modifying the responses into the signals. The sensors can be classified as optical [73,74,75], electrochemical [76,77,78], piezoelectric [79,80,81], magnetic [82,83,84], micromechanical [85,86,87] and thermal [88,89,90] sensors.

The molecularly imprinted polymer based sensors are currently a skilled and cost-effective approach to design synthetic recognition sites for biomolecules. Due to the ease of adaptation of molecularly imprinted polymers in sensors, practical applications has increased in various fields. Recent developments in biotechnology can provide the design of more effective, highly selective and sensitive, long-term stability and reusability, cheap, easy to prepare sensors [91,92]. Overall, these MIPs have a complementary advantage, which provides great facilities and flexibility in the design of imprinted polymer based sensors for selective and sensitive analyte recognition [93].

This review was regulated by different sensing techniques which include optical sensing, electrochemical sensing and piezoelectric sensing and presenting completely analyzed for each molecularly imprinted polymer based methods in diverse and recent medical applications. 

## 4. Recent Advances in Molecularly Imprinted Polymer Based Sensors

The need to develop in diagnosis has led to the use of polymers in medical applications. The use of polymers presents unique prospects for the development of global health. Furthermore, polymers are finding excellent opportunities in the medical applications, including diagnostics, point of care devices, theranostics, bio-imaging, drug delivery and cancer therapy. Because their large-scale production is claimed to be cheaper than the preparation of antibodies, MIPs are expected to be used as recognition elements in the decentralized medical diagnostics. MIPs based sensors have wide applications for the detection of biomolecules in medical diagnostics [94,95,96,97].

### 4.1. Optical Sensors

Optical sensors focus on to measure the optical characteristics change of the transducer surface when the target and recognition element form a complex [98]. These sensors can be divided into two groups. In the direct optical sensors, signal generation bases on the development of a complex on the transducer surface. The indirect optical sensors are mostly designed with a number of labels to detect the binding events and amplify the signal [99]. In the literature and the market, there are multiple optical sensors, including optrode-based fiber, evanescent wave fiber, time-resolved fluorescence, resonant mirror, interferometric and surface plasmon resonance [100,101,102,103]. Their detection window is so versatile and they sense multiple types of biomolecules from physiological and biological specimens [104].

**Figure 3:**
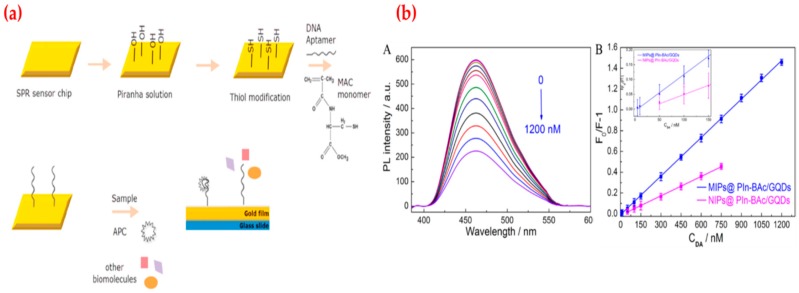
(**a**) Scheme of SPR sensor for human protein A detection and (**b**) fluorescence emission spectra with different dopamine concentration (A), the calibration curve (B). Republished with permission from [105,107]; permission conveyed through the Copyright Clearance Center, Inc.

Recently, Koyun et al. prepared an aptamer based surface plasmon resonance (SPR) sensor using different chemical modification steps for human activated protein C (APC) detection (Figure 3a). Protein C is a clotting inhibitor and the most important anticoagulant and antithrombotic agent of the human coagulation mechanism. They first characterized SPR sensors by different analyses and then tested affinity and selectivity to determine kinetic parameters. They showed the SPR sensor detected human APC in the range from 0.005 μg/mL to 4.0 μg/mL with a low limit of detection and quantification values (1.5 ng/mL and 5.2 ng/mL) [105]. Saylan et al. reported hemoglobin-imprinted sensor results for hemoglobin detection using an SPR system. Hemoglobin is an iron carrying protein in erythrocytes and also an essential element to transfer oxygen from the lungs to the tissues. Abnormalities in hemoglobin concentration are closely correlated with health status and many diseases. The hemoglobin-imprinted SPR sensor was characterized first and followed by detecting hemoglobin from 0.0005 mg/mL to 1.0 mg/mL concentration. They also calculated the lowest real-time detection performance of hemoglobin-imprinted SPR sensor as 0.00035 mg/mL of hemoglobin. After the selectivity, reusability and storage stability performances, they claimed that SPR sensor can be an alternative to current hemoglobin detection methods and the hemoglobin-imprinted SPR sensor can be combined with portable detectors to improve its access to binding sites and also tailored to detect other biomolecules [106]. Zhou et al. promoted a fluorescence sensor to determine dopamine using graphene quantum dots and composite material. Dopamine is one of the most significant neurotransmitters in the central nervous system and plays an essential role in cellular metabolism and hormonal systems. They observed that when the dopamine is added to the detection system, it causes fluorescence quenching due to the covalent binding. They calculated the limit of detection value as 2.5 × 10^−9^ M with the range of 5 × 10^−9^–1.2 × 10^−6^ M of dopamine (Figure 3b). Finally, they carried out selectivity and real-sample studies for the dopamine determination in human samples [107]. 

**Figure 4:**
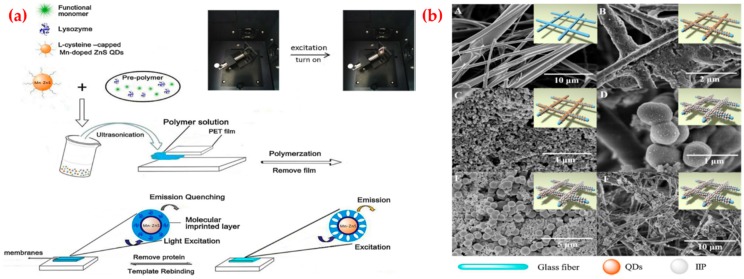
(**a**) Synthesis of quantum dots embedded imprinted membranes and (**b**) scanning electron microscope images of paper and quantum dots based ion-imprinted polymers: (A) the bare, (B) quantum dots grafted on the glass fiber, (C) quantum dots; (D) ion-imprinted polymer; (E) ion-imprinted polymer bonded with the quantum dots; (F) ion-imprinted polymer and quantum dots bound on the glass fiber. Republished with permission from [108,110]; permission conveyed through the Copyright Clearance Center, Inc.

Zhang et al. adapted a fluorescent membrane using manganese-doped quantum dots to detect lysozyme (Figure 4a). The abnormal level of lysozyme in serum and body fluid is associated with many diseases, such as leukemia, renal diseases, conjunctivitis and meningitis. They mentioned that the fluorescent quantum dots embedded membrane, which enables the optical readout characteristic and high selectivity within a membrane. They first optimized the experiment conditions and calculated the detection limit as 10.2 nM with a range from 1.0 × 10^−7^ to 1.0 × 10^−6^ M. They also calculated the recoveries of the imprinted membrane for lysozyme determination in real samples as 93–103% [108]. Göçenoğlu et al. published a study about imprinted nanoparticle based SPR sensor system for uric acid recognition. Uric acid is the end product of purine metabolism in the human body and it associates with several diseases including hyperuricemia and hypouricemia. They synthesized uric acid-imprinted nanoparticles by emulsion polymerization and then characterized by different analysis. After that, they prepared the SPR sensor modifying this uric acid-imprinted nanoparticles. They determined the sensing ability of the uric acid-imprinted SPR sensor applying uric acid solutions in different concentrations. At last, they calculated the limit of detection and quantification values as 0.247 mg/L and 0.825 mg/L [109]. Qi et al. proposed ion-imprinted polymers based microfluidic sensor for copper and mercury ions detection. Mercury is a highly toxic and hazardous heavy metal pollutant which can cause motion disorders and coronary heart disease. Copper is an essential trace element and closely related to human health and cause the burden of the liver and other organs, and result in metabolic disorders, liver cirrhosis and other diseases. They activated the surface of the paper with quantum dots through amino processing and ion-imprinted polymers formation and characterized surface morphology by scanning electron microscope analysis (Figure 4b). They reported that the copper- and mercury-imprinted microfluidic sensor showed high linearity from 0.11 g/L to 58.0 g/L and 0.26–34.0 g/L with a low limit of detection values and also claimed that their system lets for copper and mercury ions simultaneous detection [110].

Dibekkaya et al. provided a cyclic citrullinated peptide antibody-imprinted SPR sensor for antibody detection. Cyclic citrullinated peptide antibodies are helpful in the diagnosis of rheumatoid arthritis, which is an autoimmune disease with common chronic joint inflammation. For this purpose, they first synthesized a pre-complex by interacting acrylamide monomer with cyclic citrullinated peptide antibody and then prepared an antibody-imprinted SPR sensor by reacting with this pre-complex, crosslinker and initiator/activator pair. They performed the characterization and kinetic studies of the SPR sensor. They also investigated the selectivity performance of the sensor employing other similar structure proteins [111]. Prostate specific antigen-imprinted SPR sensor was originated for prostate specific antigen detection by Ertürk et al. Prostate specific antigen is an important biomarker for diagnosis and prognosis of prostate cancer. They performed with prostate specific antigen solutions in the range of 0.1–50 ng/mL with a low detection limit value (91 pg/mL). They evaluated ten clinical samples and presented 98% concurrence between the results found by enzyme-linked immunosorbent assay without important differences [112]. Click chemistry for glycoprotein selective surfaces fabrication was described by Stephenson-Brown et al. Many glycoproteins are intimately linked to the onset and progression of numerous heritable or acquired diseases of humans. They prepared imprinted surfaces that contain glycoprotein recognition nanocavities. These surfaces have high binding affinities (nM levels) and selectivities (thirty fold) for prostate specific antigen with respect to other glycoproteins. They claimed that this sensor can be applied in complex biological samples and be recycled multiple times with no performance lost [113].

### 4.2. Electrochemical Sensors

The electrochemical sensors are one of the most common sensor types because of the crucial advantages such as size, portability and economic features. These features enable the electrochemical sensors to be used as point-of-care devices by the patients themselves at home or in a doctor’s clinic and also makes the electrochemical sensors suitable applicants for sensing applications [114].

**Figure 5:**
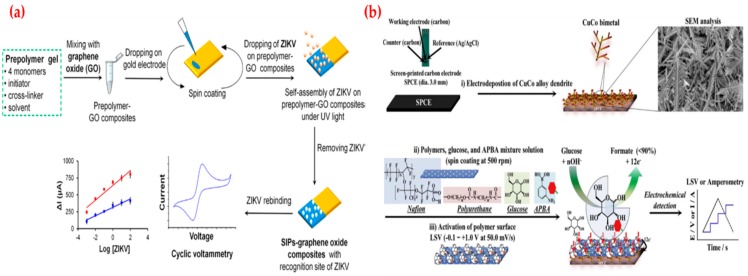
(**a**) Schematic representations of the virus-imprinted sensor and (**b**) glucose-imprinted sensor. Republished with permission from [115,116]; permission conveyed through the Copyright Clearance Center, Inc.

Recently, Tancharoen et al. developed an electrochemical sensor based on graphene oxide polymers imprinted for Zika virus detection. Zika virus is a member of the *Flaviviridae* virus family and infected individuals typically develop a mild fever, red eyes, a skin rash, conjunctivitis, muscle and joint pain, malaise, headache and then serious problems. As depicted in Figure 5a, the sensor was applied to detect virus to measure changes in the signal by changing virus concentrations in both buffer and serum solutions. They compared the detection limit value of this sensor with a commercial method and observed that it was similar to the reverse transcription [115]. Cho et al. developed a non-enzymatic sensor depending on the direct oxidation of glucose on a glucose-imprinted polymer to manage the glucose level of diabetes patients. Diabetes caused by prolonged hyperglycaemia and its complications are emerging as a serious concern; hence, treatments of diabetes have focused on the prevention and delay of chronic disorders. They first characterized the glucose-imprinted polymer coated bimetal sensor by employing analytical methods and then used in a wide range (1.0 μM–25.0 mM) with a low detection limit value (0.65 μM) (Figure 5b). They claimed the responses of analogs (dopamine, uric acid, ascorbic acid, acetaminophen, and L-cysteine) were not detected. They also showed that the performance of the sensor for glucose determination in the artificial and whole blood samples [116].

**Figure 6:**
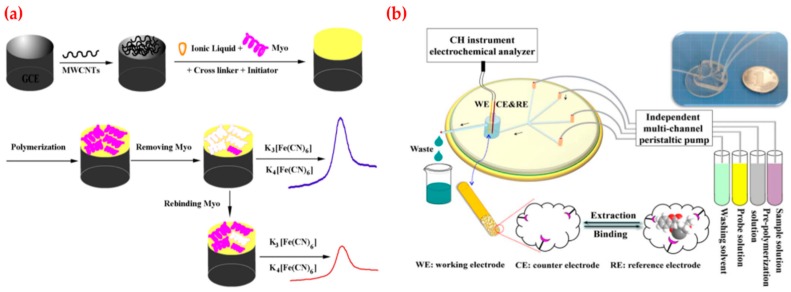
(**a**) Myoglobin-imprinted sensor and (**b**) structure, photo and application of the microfluidic sensor. Republished with permission from [117,118]; permission conveyed through the Copyright Clearance Center, Inc.

Wang et al. fabricated an imprinted polymer based electrochemical sensor for myoglobin detection (Figure 6a). Myoglobin is a hemeprotein with oxygen-binding properties and used as a biomarker for the diagnosis of acute myocardial infection. Their results demonstrated that the electrochemical sensor had high sensitivity and selectivity. They obtained an oxidation peak current at the potential of ~0.3 V that related to the concentration of myoglobin in a different range (60.0 nM–6.0 μM) with a low detection limit (9.7 nM). They also applied this electrochemical sensor to determine myoglobin amount in spiked plasma where it displayed average recoveries of 96.5% [117]. As shown in Figure 6b, Liu et al. also established the electrochemical sensor by combining molecular imprinting method with microfluidic chip and used for therapeutic drugs detection. Therapeutic drug monitoring is essential in clinical drug therapy, which aims to guarantee the effectiveness of drug and meanwhile avoid its adverse effect. They performed the characterization experiments by using cyclic voltammetry and electrochemical impedance spectroscopy and applied the linearity of the method in the range of 5 × 10^−6^–4 × 10^−4^ M. In addition, the linearity of gate effect was found as 2 × 10^−11^–4 × 10^−9^ M with a low limit of detection value (8 × 10^–12^ M), which was capable of clinical assay limits [118]. 

**Figure 7:**
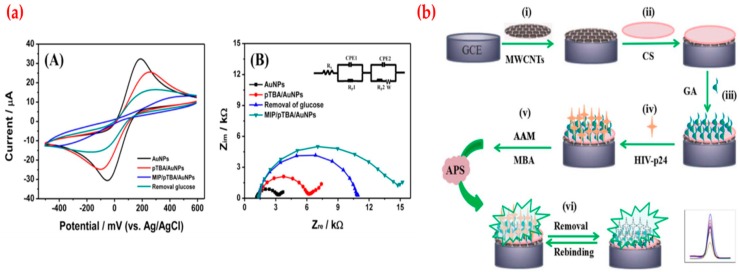
(**a**) Cyclic voltammetry curves (A) and Nyquist plots recorded for polymers (B) and (**b**) electron microscope image (A) and the size distribution (B), dark field electron image (C) and element mapping images of quantum dots (D-H). Republished with permission from [119,120]; permission conveyed through the Copyright Clearance Center, Inc.

A potentiometric sensor was developed for the detection of glucose by Kim et al. They first characterized the sensor by using cyclic voltammetry, electrochemical impedance spectroscopy, X-ray photoelectron spectroscopy, and atomic force microscope (Figure 7a) and obtained different responses at the range of 3.2 × 10^−7^ to 1.0 × 10^−3^ M with a low limit of detection value (1.9 × 10^−7^ M). They also checked the responses of the glucose sensor in real samples (saliva and blood) [119]. Ma et al. produced a sensor for the determination of human immunodeficiency virus (HIV) p24 using multi-walled carbon nanotube modified glassy carbon electrode (Figure 7b). Acquired immune deficiency syndrome (AIDS), a severe communicable immune deficiency disease caused by a retrovirus-the HIV, considered a serious pandemic and actively spread all over the world. They demonstrated that the electrochemical sensor has specific recognition ability to HIV p24 and detected the range from 1.0 × 10^−4^ ng/cm^3^ to 2.0 ng/cm^3^ with a low detection limit value (0.083 pg/cm^3^) and claimed that the electrochemical sensor had a high selectivity and stability and also it was successfully employed for the determination of HIV p24 in serum samples [120]. 

**Figure 8:**
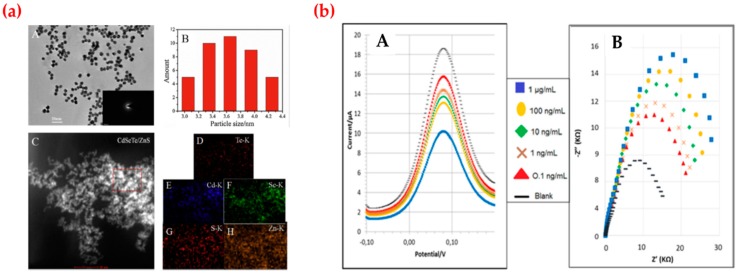
(**a**) Diagram for fabrication of the sensor and (**b**) differential pulse voltammograms (A) and Nyquist plots of creatinine solutions (B). Republished with permission from [118,119]; permission conveyed through the Copyright Clearance Center, Inc.

Wang et al. designed an electrochemical luminescence sensor based on core-shell quantum dots and imprinted composites modified glass carbon electrode for dopamine determination. After the preparation step, they first characterized core-shell quantum dots by several analyses (Figure 8a) and detected dopamine in the concentration range from 1 × 10^-14^ to 2.5 × 10^-12^ mol/L with the low limit of detection value (3.3 × 10^-15^ mol/L). They also employed this sensor to detect dopamine in the serum sample [121]. Diouf et al. developed a sensor based on imprinted screen-printed gold electrodes for creatinine detection. Creatinine is the end product of creatine metabolism in the skeletal muscles to the release of energy and is removed from the body by renal excretion at a relatively constant rate. They showed that their results indicated that the imprinted polymer based sensor had a selective detection capability for creatinine and other structurally similar compounds could not be identified. They also tested the molecularly imprinted polymer based sensor with different creatinine urine levels. As shown in Figure 8b, they calculated the detection limit value as 0.016 ng/mL and 0.081 ng/mL with a range from 0.1 ng/mL to 1 μg/mL for different techniques [122].

An imprinted electrochemical sensor for naloxone detection was prepared using screen-printed carbon electrode modification by Lopes et al. Naloxone is a morphine derivative and a specific opioid antagonist, which has high affinity for opiate receptors without activating then. They modified the carbon electrode with multi-walled carbon nanotubes for sensitivity improvement. They showed that the electrochemical sensor has a linear relation between peak intensity and naloxone concentration (0.25–10.0 µM) with a limit of detection and quantification values of 0.20 µM and 0.67 µM, respectively. They also confirmed the applicability of the electrochemical sensor in urine and human serum [123]. Nguy et al. reported a study about the detection of sarcosine. The increased concentration of sarcosine, a methylated derivate of the amino acid glycine, in urine sediments obtained from patients suffering from metastatic prostate cancer. They deposited the sarcosine-imprinted poly-aminothiophenol layers by electropolymerization onto screen-printed gold electrodes and reached the detection limit below 1 nM. Their sensor system displayed a high reproducibility, good stability, and low cross-selectivity towards other similar biomolecules [124]. Smolinska-Kempisty et al. presented a potentiometric sensor for cocaine detection relied on imprinted nanoparticles. Cocaine is one of the most widely used recreational drugs in the world with the number of millions of users among the different aged population. They selected four compositions and used two protocols. They reported that there was a good affinity for cocaine with dissociation constants between 0.6 nM and 5.3 nM. They examined the different type of cocaine in the body and observed that the sensor had a capability of cocaine detection in serum samples in the range of 1 nM and 1 mM of cocaine [125].

### 4.3. Piezoelectric Sensors

One of the members of piezoelectric sensors is the quartz crystal microbalance (QCM) sensor, which get the attention of researchers owing to its advantages such as portability, high specificity and stability and also simplicity. The quartz crystal microbalance sensors allow the monitoring of interactions by employing an oscillating crystal with the biomolecules immobilized on its surface. The binding reaction associate with an increased mass that results in a decrease of the oscillating frequency. The combination of QCM sensors with template molecule memories having MIPs through the pre-recognition provides affinity toward the template, highly selective binding sites and more sensitive sensing systems based on homogeneity in a larger number of recognition sites [126,127,128,129]. 

**Figure 9:**
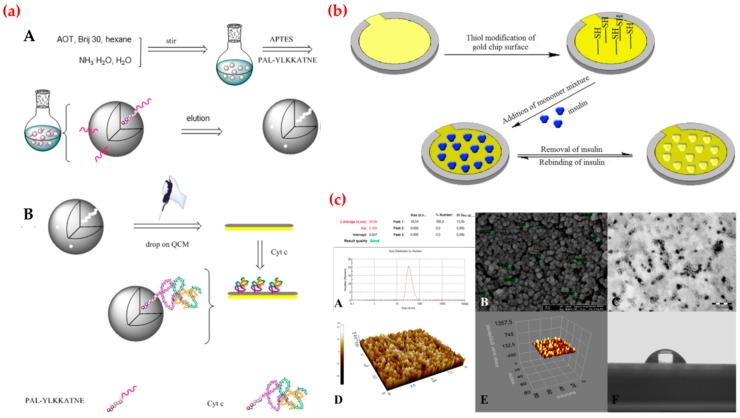
(**a**) Production of imprinted polymer (A) and sensor (B); (**b**) scheme of the insulin-imprinted sensor preparation and (**c**) characterization results: zeta-size (A), scanning (B) and transmission electron microscope (C) images of cannabinoid-imprinted nanoparticles and atomic force microscope (D), ellipsometer (E) and contact angle images of cannabinoid-imprinted sensor (F). Republished with permission from [130,131,135]; permission conveyed through the Copyright Clearance Center, Inc.

Recently, Ma et al. fabricated a QCM sensor to detect cytochrome c (Figure 9a). Cytochrome c is a heme-containing electron carrier of the mitochondrial respiratory chain. They performed real-time cytochrome c detection with the range from 0.005 μg/mL to 0.050 μg/mL and calculated detection limit value as 3.6 ng/mL. They reported that the cytochrome c-imprinted sensor exhibited high sensitivity and selectivity towards cytochrome c and it also could be applied for real sample studies with high reproducibility and accuracy. They claimed that the new sensor fabrication process based on epitope- imprinted polymers opens new approaches for selective biomolecule detection [130]. Kartal et al. provided a QCM sensor for insulin detection in both aqueous and artificial plasma solutions. Insulin secreted by pancreatic cells is an essential polypeptide hormone and a key regulator in glucose metabolism in blood. They modified the gold surface of the sensor by amino-acid based monomer (Figure 9b) and used affinity studies to obtain kinetic parameters. They also performed repeatability of insulin-imprinted sensor through four binding cycles. Finally, they calculated the detection limit value as 0.00158 ng/mL [131]. Yun et al. synthesized an amantadine-imprinted QCM sensor using gold nanoparticles and reduced graphene oxide. Amantadine, with a stable tricyclic amine structure, is generally used for the treatment of both influenza and Parkinson’s disease in the clinical treatment of animals and human beings. They optimized different modification steps of the sensor fabrication process and then characterized the sensor with several methods. They obtained a linear relationship with the concentration of the amantadine (1.0 × 10^−5^–1.0 × 10^−3^ mmol/L) with a low detection limit (5.4 × 10^−6^ mmol/L). They also calculated the imprinting factor for amantadine as 7.1 [132]. Qiu et al. prepared an imprinted QCM sensor for sialic acid determination in urine samples. Sialic acid, a negatively charged monosaccharide, is well known as a marker normally found in blood serum, which is expressed less frequently in diabetes patients than normal. The level of total sialic acid can reflect some dysfunction of the human body and further indicate an early stage of some cancers or cardiovascular disease. After the characterization experiments, they tested the selectivity performance of the sensor by recognition studies. They obtained a linear response in the range of 0.025–0.50 μmol/L and also calculated the detection limit as 1.0 nmol/L for sialic acid with high recovery values (87.6–108.5%) for urine sample [133]. Chunta et al. reported the production of imprinted QCM sensor that selectively binds high-density lipoprotein and serves as an artificial and biomimetic sensing material. A decreased blood level of high-density lipoprotein is one of the essential criteria in diagnosing metabolic syndrome associated with the development of atherosclerosis and coronary heart disease. They analyzed the performance of the sensor in a dynamic detection range of 2–250 mg/dL and examined the selectivity experiments using phospholipid, triglyceride, free cholesterol, cholesterol ester, and apolipoprotein. They claimed that this sensor exposed the high recovery rates (94–104%) and displayed a correlation with the standard in clinical tests [134]. Battal et al. produced a QCM sensor for different synthetic cannabinoids detection. Cannabinoids are chemical compounds that main active component of cannabis. They bind to cannabinoid receptors in the brain and were originally developed as therapeutic agents in the treatment of pain. They first prepared synthetic cannabinoid-imprinted nanoparticles and then characterized these nanoparticles by various analyses (Figure 9c). After that, they modified a gold surface of the sensor with these nanoparticles and used for kinetic studies. They found the limit of detection values as 0.3, 0.45, 0.4 and 0.2 pg/mL for different cannaboids. They reported that the sensors showed were highly sensitive and selective in a wide concentration range (0.0005–1.0 ng/mL) in both aqueous and synthetic urine samples [135].

Bakhshpour et al. carried out protein C detection in serum by employing a QCM sensor. They used a micro-contact imprinting method to prepare protein C-imprinted sensor. They investigated protein C detection in a range of 0.1–30 µg/mL. The limit of detection value was also calculated as 0.01 µg/mL. Finally, they examined the repeatability of the sensor with five adsorption-desorption cycles [136]. Çiçek et al. prepared a QCM sensor to detect bilirubin in plasma. Bilirubin is a tetrapyrrolic yellow compound, formed as an end product of heme in blood and transported to hepatocytes via albumin (as a water-soluble complex) for further conjugation with glucuronic acid. They polymerized the bilirubin-imprinted nanofilm on the sensor surface and characterized by several measurements. They examined the kinetic and affinity properties of the bilirubin-imprinted sensor and calculated the detection limit value as 0.45 µg/mL [137]. The QCM sensor was provided for 17β-estradiol monitoring through the immobilization of the nanoparticles by Özgür et al. The 17β-estradiol, natural steroid hormone, is one of the most potent endocrine disrupting compounds. After the characterization experiments, their kinetic results displayed that the sensor showed high selectivity and sensitivity for 17β-estradiol in a range of 3.67 nM-3.67 pM. They found the values of the limit of detection and quantification values as 613 fM and 2.04 pM, respectively [138]. 

## 5. Conclusions and Outlook

Sensors are widely used in biomedical research, health care, and pharmaceutical research to detect biomolecules for the diagnosis of various diseases. In this review, the medical applications of sensors prepared by molecularly imprinted polymers were summarized. The main challenge in the medical analysis is to find unique biomolecule and early determination of their quite low concentrations within a complex matrix, which is very important for the diagnosis of certain diseases. Molecularly imprinted polymer based sensing is thus attractive because of its specificity, selectivity, cost efficiency, physical and chemical stability with respect to the biomolecules, ease of preparation and applicability of the method for diverse biomolecules. According to the literature the attempts on imprinted polymer based sensors are able to provide a low limit of detection values, which are close to the conventional methods. These values are improved with the use of several polymeric or gold nanoparticles and also the computer-based determination of most suitable functional ligands and their possible combination ratios provide much better interaction with the analyte with improved specificity. Molecularly imprinted polymers based sensor systems are expected to grow continuously and rapidly in the medical applications as well as other platforms. Sensor technology has still developed to a point where the biomolecule testing has been moved to near patient diagnostic methods from large centralized high throughput laboratories. In future these sensors can be developed in the form of hand held devices where the patient can test and see the results by her/himself without medical assistance. This type of sensors will revolutionize the health care industry by reducing the treatment cost and by improving the clinical outcomes.

## Abbreviations

Acquired immune deficiency syndrome	AIDS
Acrylamide	AAM
Activated protein C	APC
Aminophenyl- boronic acid	APBA
3-Aminopropyltriethoxysilane	APTES
Chitosan	CS
Copper	Cu
Counter electrode	CE
Cytochrome c	Cyt c
Dioctyl sulfosuccinate sodium salt	AOT
Dopamine	DA
Electrogenerated chemiluminescence	ECL
Glucose-imprinted polymer	GIP
Glutaraldehyde	GA
Gold nanoparticles	AuNPs
Graphene oxide	GO
Human immunodeficiency virus p24	HIV-p24
Ion-imprinted polymer	IIP
Limit of detection	LOD
Limit of quantification	LOQ
Lysozyme	Lys
N,N′-Methylenebisacrylamide	MBA
N-Methacryloyl-L-cysteine	MAC
Mercury	Hg
Molecularly imprinted polymers	MIPs
Molecular imprinting technology	MIT
Multi-walled carbon nanotubes	MWCNTs
Myoglobin	Myo
Non-imprinted polymers	NIPs
Poly(terthiophene)	pTBA
Quantum dots	QDs
Quartz crystal microbalance	QCM
Reference electrode	RE
Scanning electron microscope	SEM
screen printed carbon electrode	SPCE
Surface imprinted polymers	SIPs
Surface plasmon resonance	SPR
Synthetic cannabinoids	SCs
Ultraviolet	UV
Working electrode	WE
Zika virus	ZIKV
